# The two-year progression of structural and functional cerebral MRI in amyotrophic lateral sclerosis

**DOI:** 10.1016/j.nicl.2017.12.025

**Published:** 2017-12-18

**Authors:** R.A.L. Menke, M. Proudfoot, K. Talbot, M.R. Turner

**Affiliations:** a Wellcome Centre for Integrative Neuroimaging, FMRIB, Nuffield Department of Clinical Neurosciences University of Oxford, Oxford, UK; b Nuffield Department of Clinical Neurosciences, University of Oxford, Oxford, UK

## Abstract

MRI has emerged as one of several urgently needed candidate disease progression biomarkers for the neurodegenerative disorder amyotrophic lateral sclerosis (ALS), not least due to its unique ability to non-invasively assess structural and functional cerebral pathology. We sought to identify the extent of detectable change in cerebral MRI metrics over a more prolonged period.

Analysis of multi-modal MRI data was performed in a cohort of sixteen patients (13 ALS and 3 with primary lateral sclerosis) in whom it was possible to acquire six-monthly images over two years. Structural brain changes were assessed using voxel-based morphometry of grey matter and shape analysis of sub-cortical grey matter structures, tract-based spatial statistics of diffusion tensor imaging (DTI) metrics optimized for longitudinal analysis in the white matter, as well as whole brain voxel-wise statistics of DTI metrics. Changes in resting state functional MRI (rs-fMRI) were investigated via independent component and dual regression analyses of functional connectivity (FC), controlled for confounding effects of grey matter decline. Both linear changes with time and brain changes correlated with revised ALS functional rating score (ALSFRS-R) decline were studied.

Widespread and progressive reductions in grey matter were observed in the precentral gyri and posterior cingulate cortex, as well as progressive local atrophy of the thalamus, caudate, and pallidum bilaterally, and right putamen, hippocampus and amygdala. The most prominent DTI tract-based changes were in the superior longitudinal fasciculi and corpus callosum. More widespread areas of DTI changes included the thalami and caudate nuclei, hippocampi and parahippocampal gyri, insular cortices, anterior and posterior cingulate gyri, frontal operculum and cerebellum. FC decreases were noted between the sensorimotor resting state network and the frontal pole, between a network comprising both thalami and an area in the visual cortex, in relation to both time from baseline and ALSFRS-R decline. FC increases between the left primary motor cortex and left fronto-parietal network were seen for both statistical approaches.

A longer period of follow-up, though necessarily involving more slowly-progressive cases, demonstrated widespread changes in both grey and white matter structural MRI measures. The mixed picture of regional decreases and increases in FC is compatible with compensatory change, in what should be viewed as a brain-based disease characterised by larger-scale disintegration of motor and frontal projection cerebral networks.

## Highlights

• Analysis of serial MRI data (6-monthly over 2 years) was performed in ALS patients.
• Widespread progressive structural and functional brain changes were observed.
• Changes during this unprecedented study period involved basal ganglia regions.
• The results revealed novel longitudinal functional connectivity insights.

## 1 Introduction

Amyotrophic lateral sclerosis (ALS) is a uniquely destructive and typically rapidly progressive neurodegenerative disorder, with a median survival of 30 months from symptom onset. Historically defined by loss of both upper motor neurons of the corticospinal tract (CST) neurons and lower motor neurons of certain brainstem nuclei and spinal anterior horns, there is now an established clinical pathological and genetic overlap with frontotemporal dementia Turner and Swash (2015).

The causes of ALS are protean and complex, with effective therapy remaining elusive, in part due to the lack of objective biomarkers of disease activity and progression against which to assess candidates (Turner and Benatar, 2015). While the revised Amyotrophic Lateral Sclerosis Functional Rating Scale (ALSFRS-R) remains the leading outcome measure in therapeutic trials, it has significant limitations (Proudfoot et al., 2016). Advanced magnetic resonance imaging (MRI) has uniquely enabled structural and functional abnormalities of the central nervous system to be observed in ALS patients in vivo. MRI has emerged as one of several biomarker candidates that could not only assist differential diagnosis and patient stratification, but also offer a probe for the investigation of natural disease progression and the effectiveness of therapeutic approaches (Turner et al., 2011, Turner and Verstraete, 2015).

Several longitudinal MRI studies have been conducted in ALS, but the goal of MRI as a therapeutic trial endpoint requires a greater understanding of the natural history of MRI changes in ALS, as well as addressing inconsistencies in relation to clinical heterogeneity, and MRI acquisition and analysis protocols (Menke et al., 2016). In a large structural MRI study conducted by our group, analysis of diffusion tensor imaging (DTI) data showed limited increase in axial and mean diffusivity in a corpus callosum region of interest, as well as minor axial diffusivity increases in the left CST over a minimum of six months, while voxel-based morphometry (VBM) analysis revealed widespread grey matter volume decreases in motor and frontotemporal regions, thalami and caudate heads bilaterally (Menke et al., 2014). In contrast, in a study of three time points over an interval of six months, neither VBM, cortical thickness analysis nor volumetry of deep brain structures indicated progressive grey matter atrophy, while a linear decline in the DTI-derived fractional anisotropy of the CST was reported (Cardenas-Blanco et al., 2016).

In order to identify the extent of structural *and* functional MRI changes detectable over a longer period of follow-up, we performed a multimodal analysis across a cohort of patients in which it was possible to acquire six-monthly MRI data over two years.

## 2 Methods

### 2.1 Participants

ALS patients seen in a large tertiary referral clinic were offered participation in the Oxford Study for Biomarkers in MND (‘BioMOx’) between 2009 and 2013, involving a range of biomarker assessments at six-monthly intervals until physical disability or death prevented further study. Sixteen patients (six females) from a total of 95, 13 with ALS and 3 with primary lateral sclerosis (PLS) according to standard criteria (Brooks et al., 2000, Gordon et al., 2006), completed five multi-modal MRI assessments approximately every six months over a period of two years (average interval between time points was 6.4 ± 0.6, 6.7 ± 0.5, 6.5 ± 1.5, and 7.2 ± 1.6 months). All were apparently sporadic cases (i.e. no reported family history of ALS or FTD), and none fulfilled diagnostic criteria for dementia (Strong et al., 2016). Participants underwent clinical examination on the day of study, including ALSFRS-R score (0–48, with lower total reflecting higher disability). Disease duration was calculated from symptom onset to scan date in months, and the rate of progression determined by: (48 minus current ALSFRS-R) / disease duration. An upper motor neuron (UMN) clinical burden score comprised the total number of pathologically brisk reflexes on examination of 15 sites (Menke et al., 2014).

Table 1 contains detailed demographic information and clinical details for each patient. In summary, at time of the first scan the mean age was 60 ± 12 years (range 38–83), mean disease duration was 89.5 ± 90.9 months (range 17–358 months), the mean ALSFRS-R score was 34.0 ± 4.6 (range 27–43), the mean UMN score was 8.3 ± 5.4 (range 0–15), and the mean progression rate at first scan was 0.25 ± 0.15 (range 0.05–0.57). A repeated measures ANOVA with a Greenhouse-Geisser correction (IBM SPSS Statistics, Version 21) showed that mean ALSFRS-R differed significantly between time points [F(1.541, 23.108) = 46.760, *p* < 0.001]. Post hoc tests using Bonferroni correction revealed that ALSFRS-R reduced by an average of 2.6 points between scan 1 and 2 (p < 0.001), by an additional 2.4 points between scan 2 and 3 (*p* = 0.001), an additional 1.2 points between scan 3 and 4 (*p* = 0.027), and 1.8 points between scan 4 and 5 (*p* = 0.006). Normality checks were carried out on the residuals, which were approximately normally distributed (Shapiro-Wilk tests, IBM SPSS Statistics, Version 21).

**Table 1:** Demographic and clinical information.

ID	Age	Gender	Handedness	Side of onset	Diagnosis	Disease duration	UMN	ALSFRS-R	Progression rate
P01	69	M	R	RUL	ALS	32, 39, 46, 53, 59	5, 6, 6, 5, 5	33, 32, 27, 27, 26	0.46, 0.41, 0.46, 0.40, 0.37
P02	67	F	R	BO	PLS	63, 69, 76, 82, 88	13, 12, 12, 13, 13	33, 29, 26, 27, 23	0.24, 0.27, 0.26, 0.26, 0.29
P03	69	M	R	LUL	ALS	33, 38, 45, 51, 56	4, 4, 2, NT, NT	39, 34, 34, 31, 29	0.27, 0.36, 0.31, 0.33, 0.34
P04	48	F	R	RUL	ALS	67, 74, 80, 86, 96	5, 5, 5, 5, 5	33, 32, 29, 29, 27	0.22, 0.22, 0.24, 0.22, 0.22
P05	55	F	L	LLL	ALS	79, 85, 92, 98, 106	4, 5, 3, 3, 4	37, 35, 33, 31, 30	0.14, 0.14, 0.14, 0.17, 0.17
P06	56	F	R	LLs	ALS	65, 72, 80, 86, 93	3, 3, 1, 3, NT	27, 24, 23, 22, 20	0.32, 0.33, 0.31, 0.30, 0.30
P07	38	M	R	RUL	ALS	17, 24, 30, 36, 42	10, 10, 10, NT, NT	41, 36, 34, 34, 32	0.41, 0.51, 0.46, 0.39. 0.38
P08	69	M	R	RLL	PLS	358, 366, 373, 384, 389	13, 13, 13, 13, 13	31, 29, 27, 25, 25	0.05, 0.05, 0.06, 0.06, 0.06
P09	49	M	R	LLL	ALS	28, 34, 41, 46, 53	15, 15, 15, 14, NT	36, 31, 27, 23, 17	0.43, 0.49, 0.52, 0.54, 0.58
P10	39	M	R	LUL	ALS	23, 28, 34, 41, 48	12, 15, 15, 15, 15	35, 32, 27, 26, 23	0.57, 0.57. 0.62, 0.54, 0.52
P11	63	M	R	LLL	ALS	75, 82, 88, 95, 102	0, 1, 1, 1, 1	30, 29, 28, 28, 27	0.24, 0.23, 0.23, 0.21, 0.21
P12	60	M	R	RUL	ALS	121, 128, 135, 141, 148	1, 1, 1, 1, 1	28, 27, 23, 23, 23	0.16, 0.16, 0.19, 0.18, 0.17
P13	83	M	R	RUL	ALS	106, 112, 119, 124, 131	4, 4, 4, 5, 5	32, 30, 29, 27, 25	0.15, 0.16, 0.16, 0.17, 0.18
P14	64	F	R	LLL	PLS	247, 254, 260, 270, 280	15, 15, 15, 14, NT	29, 28, 28, 26, 24	0.08, 0.08, 0.08, 0.08, 0.09
P15	62	F	R	LLL	PLS	86, 92, 99, 105, 112	15, 15, 15, 15, 15	37, 32, 27, 25, 25	0.13, 0.17, 0.21, 0.22, 0.21
P16	65	M	R	BO	ALS	30, 36, 43, 50, 59	13, 11, 11, NT, 10	43, 43, 43, 42, 42	0.17, 0.14, 0.12, 0.12, 0.10

Age is given years. Disease duration is given in months. RUL – right upper limb, BO – bulbar onset, LUL – left upper limb, LLL – left lower limb, LLs – lower limbs, RLL – right lower limb. UMN – Upper Motor Neuron score. ALSFRS-R – Revised ALS Functional Rating Scale. NT – not tested. Results for different time points are comma separated.

Cognitive assessment (see Table 2) was undertaken using the revised Addenbrooke's Cognitive Examination Score (ACE-R, maximum score 100, with subscores for verbal fluency for ‘p words in 1 min’, ‘animals in 1 min’, and a memory task, each scored 0–7) (Mioshi et al., 2006).

**Table 2:** Basic cognitive testing information.

ID	ACE%	VF- P words	VF- animals	Memory recall
P01	NT, 88, 90, 88, NT	NT, 4, 4, 4, NT	NT, 5, 7, 7, NT	NT, 4, 5, 3, NT
P02	NT, 100, 98, 100, NA	NT, NA, NA, NA, NA	NT, NA, NA, NA, NA	NT, 7, 7, 7, NA
P03	93, 97, 94, 98, 95	5, 6, 5, 6, 5	6, 7, 7, 7, 6	6, 6, 7, 7, 7
P04	NT, 98, 97, 97, 94	NT, 6, 5, 5, 6	NT, 7, 7, 7, 7	NT, 7, 7, 6, 5
P05	NT, 100, 100, 100, 100	NT, 7, 7, 7, 7	NT, 7, 7, 7, 7	NT, 7, 7, 7, 7
P06	99, 100, 99, 100, 100	7, 7, 7, 7, 7	7, 7, 7, 7, 7	7, 7, 7, 7, 7
P07	91, 97, 94, 100, 100	6, 7, 6, 7, 7	7, 6, 6, 7, 7	7, 6, 6, 7, 7
P08	78, 94, 82, 83, 83	4, 5, 5, 6, 4	7, 5, 6, 6, 5	2, 7, 2, 7, 7
P09	NT, 93, 97, NT, NT	NT, 5, 5, NT, NT	NT, 5, 6, NT, NT	NT, 7, 7, NT, NT
P10	NT, NT, 100, 99, 97	NT, NT, 7, 6, 5	NT, NT, 7, 7, 7	NT, NT, 7, 7, 7
P11	NT, 84, 87, 86, 81	NT, 6, 6, 6, 5	NT, 6, 4, 6, 5	NT, 0, 0, 0, 2
P12	NT, 95, 94, 93, 98	NT, 7, 6, 6, 6	NT, 7, 5, 7, 7	NT, 6, 7, 6, 7
P13	NT, 91, 89, 86, 86	NT, 4, 4, 4, 4	NT, 4, 5, 4, 4	NT, 6, 5, 5, 3
P14	90, 82, 98, 98, 98	4, NA, NA, NA, NA	3, NA, NA, NA, NA	4, 7, 7, 7, 7
P15	96, 98, 96, NT, 97	7, 6, 7, NT, 6	6, 7, 7, NT, 7	7, 7, 6, NT, 7
P16	98, 98, 98, 100, 98	6, 6, 6, NA, 7	7, 6, 6, NA, 6	7, 7, 7, 7, 6

ACE – Revised Addenbrooke's Cognitive Examination (total score is 100%, higher scores indicate better cognitive functioning). VF – Verbal Fluency. NT – not tested. NA – patient was unable to perform. Scores for different time points are comma separated.

Ethical approval for all procedures was obtained (South Central Oxford Ethics Committee: 08/H0605/85), with written informed consent obtained from all participants.

### 2.2 Image acquisition

Scans were performed at the Oxford Centre for Clinical Magnetic Resonance (OCMR) using 3T Siemens Trio scanner (Siemens AG) with a 12-channel head coil. For each subject, T1-weighted images were obtained using a 3D Magnetization Prepared-Rapid Acquisition Gradient Echo (MP-RAGE) sequence (192 axial slices, flip angle: 8°, 1 × 1 × 1 mm^3^ voxel size, TE/TR/TI = 4.7 ms/2040 ms/900 ms). Acquisition time for the MP-RAGE image was 6 min. Whole-brain DTI images were acquired using an echoplanar imaging sequence (60 isotropic directions; b value = 1000 s/mm^2^; echo time/repetition time = 94 ms/10000 ms; 2 × 2 × 2 mm^3^ voxel size; 65 slices). In addition, four images without diffusion weighting were acquired. Whole-brain functional imaging at rest (rs-fMRI) was performed using a gradient echo EPI sequence (TR/TE = 3000/28 ms, flip angle = 89°, 3 mm isotropic resolution, 6 min acquisition time). For consistency, subjects were instructed to close their eyes throughout, but to remain awake. Furthermore, a field map was acquired using a gradient echo imaging sequence (2 × 2 × 2 mm^3^ voxel size; 65 slices; echo time 1/echo time 2/repetition time = 5.19 ms/7.65 ms/655 ms) to account for distortions present in the DTI and functional MRI data caused by field inhomogeneities.

### 2.3 Image analysis

All images were analyzed using tools from the FMRIB Software Library (FSL) (Smith et al., 2004).

#### 2.3.1 Intra-subject midway space registration

Before conducting the analyses described in the following paragraphs, each patient's MRI images for all time points were registered to a common ‘midway’ space in order to avoid potential registration-related bias. First, each patient's structural images were brain-extracted (Smith, 2002), linearly registered to the structural image from the second follow-up (Fu2) visit, and averaged to create a subject specific template in a common space.

All original structural images were then linearly registered to this template and the command ‘midtrans’ (implemented in FSL) was run utilizing the subject specific template in Fu2 space, as well as the transformation matrices for the registration of the structural images for every time point to this template, thus calculating a transformation matrix for the registration of the template into the midway space between all five structural images.

Lastly, the structural-to-template and template-to-midway space transformations were concatenated and applied in order to register the original structural images for each time point into the subject specific midway space for subsequent analysis. DTI and rs-fMRI data in midway space were obtained by registration of the relevant data for each subject/time point for both modalities to the respective original structural images first, followed by application of the structural-to-midway space transformation.

#### 2.3.2 Voxel-based morphometry (VBM)

T1-weighted MP-RAGE data in midway space was analyzed with FSL-VBM, a voxel-based morphometry style analysis (Good et al., 2001, Smith et al., 2004, Douaud et al., 2007). First, images were brain-extracted (Smith, 2002). Next, tissue-type segmentation was carried out using FAST4 (Zhang et al., 2001). The resulting grey matter partial volume images were then aligned to MNI152 standard space using the affine registration tool FLIRT (Jenkinson and Smith, 2001, Jenkinson et al., 2002), followed by nonlinear registration using FNIRT (Anderson et al., 2007). The outputted images were averaged to create a symmetric, study-specific template, to which the grey matter images in midway space were then non-linearly re-registered. The registered partial volume images of all subjects were then multiplied by the Jacobian of the warp field (‘modulation’) to correct for local expansion or contraction. The modulated segmented images were then smoothed with an isotropic Gaussian kernel with a sigma of 3 mm.

#### 2.3.3 Volumetric and shape analysis of subcortical grey matter structures (FIRST)

The putamen, caudate, pallidum, thalamus, amygdala and hippocampus were segmented from each participant's MPRAGE image using FMRIB's Integrated Registration and Segmentation Tool (FIRST). The results of the subcortical segmentation were carefully examined to ensure accuracy of the results. Vertex analysis was then performed using FIRST in a mode of operation that aims to assess group differences on a per-vertex basis (the meshes were reconstructed in native space).

#### 2.3.4 Diffusion Tensor Imaging (DTI) general pre-processing

DTI data from three subjects (2 ALS, 1 PLS) were necessarily removed from the analysis due to spiking artefacts noted during quality control procedures. Each remaining subject's DTI scans for all five time points were corrected for head motion and eddy currents and then brain-extracted to remove any non-brain voxels. To correct for B_0_ inhomogeneity and to unwarp scans, field map correction was performed with FUGUE, part of FSL. Fractional anisotropy (FA), mean diffusivity (MD), axial diffusivity (AD, eigenvector L1) and L2 and L3 maps were created using DTIFIT by applying a diffusion tensor model to each voxel (Behrens et al., 2003). Radial diffusivity (RD) maps were created by averaging the L2 and L3 maps (RD = (L2 + L3)/2). Lastly, for each patient, all quantitative DTI maps were registered to the respective midway space for subsequent analysis.

#### 2.3.5 DTI Tract-based Spatial Statistics (TBSS) pre-processing

Each subject's five time point FA images in their respective subject specific midway space were non-linearly registered to a standard FA template (http://fsl.fmrib.ox.ac.uk/fsl/fslwiki/FMRIB58_FA), and then averaged to create a study-specific template to which each subject's/time point's FA maps were then non-linearly registered. Next, the mean FA image was created and thinned to create a mean FA skeleton (Smith et al., 2006), which represents the centers of all tracts common to all data. Each subject's aligned FA data from the first time point was then projected onto this skeleton. The same operations that were used to register the individual first time point FA images to the study-specific template and project FA values onto the mean FA skeleton were subsequently applied to the FA data for all follow up scans (to ensure that the same voxels were compared during statistical analysis), and were then also applied to each subject's MD, L1, and RD images for every available time point.

#### 2.3.6 Whole-brain analyses of DTI metrics

To test whole-brain DTI changes over time each subject's five time point DTI maps in MNI space derived from the TBSS pre-processing pipeline were fed into the statistical analyses detailed below instead of the skeletonized versions, which focus solely on white matter tract centers.

#### 2.3.7 Resting state functional MRI (rs-fMRI)

Rs-fMRI analysis was performed using independent component analysis (ICA) as implemented in the FSL tool MELODIC (Beckmann and Smith, 2004). The ICA approach was favoured over seed-based correlation analysis because it enables automated isolation of resting-state brain networks (RSNs) where individual areas in a given network are tightly functionally connected and avoids seed selection bias (Cole et al., 2010). Individual preprocessing consisted of motion correction, brain extraction, unwarping using fieldmap data, spatial smoothing using the Gaussian kernel of FWHM of 6 mm, and high-pass temporal filtering of 150 s. To correct for motion, physiological noise and other artefacts, a previously described ICA-based de-noising approach was used (Griffanti et al., 2014). After performing participant-level ICA with automated dimensionality estimation, the FSL tool FIX (Salimi-Khorshidi et al., 2014) was used to automatically classify the obtained components into signal or noise, and the noise contribution was regressed out from the data. Subsequently, data were linearly registered to the corresponding structural image in midway space using FLIRT, optimized using Boundary-Based Registration (Greve and Fischl, 2009) and subsequently registered (via the structural image) to the MNI space using non-linear registration.

A group template of independent components (i.e., RSNs) was then generated from all participants and time points via ‘multi-session temporal concatenation’ as implemented in MELODIC (the number of output components was limited to ‘20’). The dual regression approach (Douaud et al., 2011) was then used to identify individual temporal dynamics and the associated spatial maps for all RSNs of interest. Spatial regression was performed using the spatial maps obtained from the resting state template in a general linear model (GLM) against each participant's individual fMRI data, which resulted in participant-specific time courses for each of the components. These time courses were then used in a further GLM to generate participant-specific spatial maps for each RSN.

#### 2.3.8 Statistical analyses

To assess grey and white matter decline over time, voxel-wise GLM was applied using permutation-based non-parametric testing. Linear changes with time and brain changes correlated with ALSFRS-R decline were studied. The ‘time’ eigenvector (EV) was created by subtracting each subject's disease duration (in days) for each scan by the disease duration on the day of the first scan and subsequent demeaning (values were demeaned separately for each subject). The ALSFRS-R EV was created following the same principle.

The main EV was then created by combining the resulting values for all subjects and time points in an order that corresponded to the subject/time point order in the 4D image files.

16 additional EVs (one for each subject) were included that specified the input for a specific patient (i.e., one extra column per subject; each contained five ‘1’s and 75 zeros for the 15 other patients' five time points; similar to the ‘two groups, paired’ option in the GLM setup application available in FSL). No confound regressors were included in the design.

Results were considered significant for *p* < 0.05, after correction for multiple comparisons (family wise error, FWE) using the threshold-free cluster enhancement (TFCE) approach (Smith and Nichols, 2009).

Similarly, rs-fMRI functional connectivity (FC) was investigated via independent component and dual regression analyses, with an additional VBM voxelwise regressor included in the voxel-wise GLM for the FC analysis to control for potential confounding effects of grey matter decline.

## 3 Results

### 3.1 Grey matter changes

**Fig. 1:**
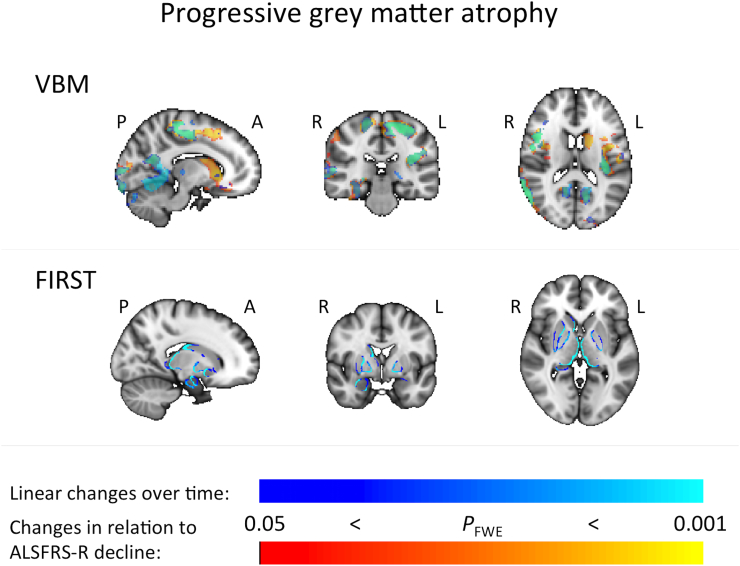
Progressive grey matter decline over time (blue), overlaid (semi-transparent) onto changes in relation to ALSFRS-R decline (red-yellow), so that overlapping areas appear in green color. Top: Results of whole brain voxel-based morphometry analyses (‘VBM’). Bottom: Results of shape analysis of sub-cortical grey matter structures (‘FIRST’). P – posterior, A – anterior, R – right, L – left. (For interpretation of the references to color in this figure legend, the reader is referred to the web version of this article.)

VBM analysis revealed widespread grey matter decline, covering precentral gyri and posterior cingulate cortex for both statistical approaches. Shape analysis of sub-cortical structures revealed nearly identical patterns of progressive local atrophy of the thalamus, caudate, and pallidum bilaterally, and for the right putamen, hippocampus and amygdala for both statistical approaches (Fig. 1).

### 3.2 White matter tract changes

**Fig. 2:**
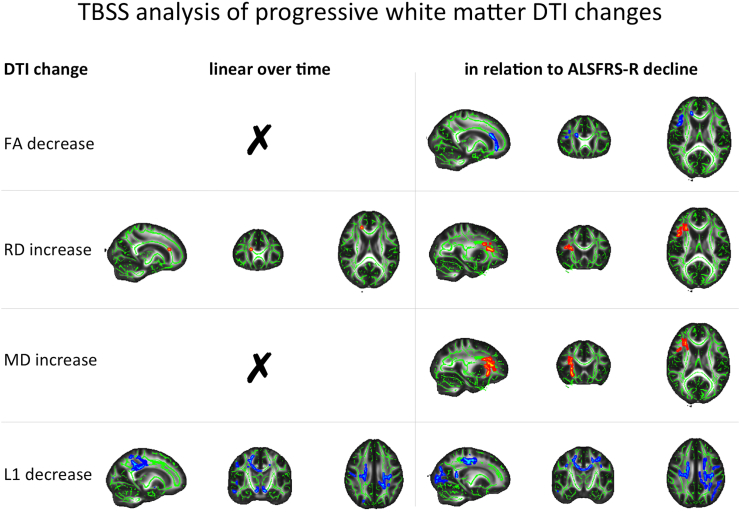
Progressive white matter decline over time (left panel) and in relation to ALSFRS-R decline (right panel). FA – fractional anisotropy, RD – radial diffusivity, MD – mean diffusivity, L1 – axial diffusivity. For better visibility, areas of significant results were thickened using tbss_fill. Decreases are illustrated in blue, increases are shown in red-yellow. Changes are overlaid onto the mean FA skeleton from the first time point (green) and the FMRIB58_FA_1mm.nii.gz template in MNI space (greyscale). (For interpretation of the references to color in this figure legend, the reader is referred to the web version of this article.)

Radial diffusivity in the right superior longitudinal fasciculus was increased in relation to both time from baseline and ALSFRS-R decline, as well as in the right forceps minor over time and in the right inferior fronto-occipital fasciculus with decreasing ALSFRS-R. Both statistical approaches demonstrated widespread axial diffusivity decreases bilaterally in the CSTs, forceps major, superior longitudinal fasciculi (particularly in the temporal parts), as well as in the body and splenium of the corpus callosum. Additionally, axial diffusivity was found to decrease over time in the cerebral peduncle level corticospinal tract and in the anterior thalamic radiation. In relation to ALSFRS-R decline, FA decreases were seen in the right superior longitudinal fasciculus, right genus of the corpus callosum and right forceps minor, with MD increases in the right superior longitudinal fasciculus, right inferior fronto-occipital fasciculus, and right anterior thalamic radiation (Fig. 2).

### 3.3 Whole-brain DTI changes

**Fig. 3:**
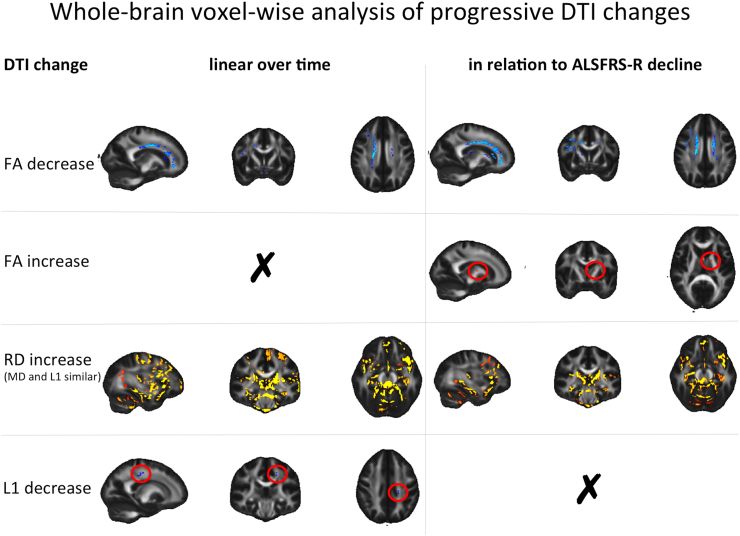
Progressive change of whole-brain diffusion tensor imaging metrics over time (left panel) and in relation to ALSFRS-R decline (right panel). FA – fractional anisotropy, RD – radial diffusivity, L1 – axial diffusivity. Decreases are illustrated in blue; increases are shown in red-yellow. Changes are overlaid onto the FMRIB58_FA_1mm template in MNI space (greyscale) and have in part been encircled in red for improved visibility.(For interpretation of the references to color in this figure legend, the reader is referred to the web version of this article.)

The whole-brain analyses revealed FA decreases in the right superior longitudinal fasciculus, right genu of the corpus callosum, body of corpus callosum, right anterior corona radiata, and the right frontal medial cortex, both in relation to time from baseline and ALSFRS-R decline. FA decreases were seen in the left anterior corona radiata, left middle frontal gyrus, left superior cingulate gyrus, right thalamus and in the right inferior frontal gyrus. Widespread, co-localized increases of radial, axial, and mean diffusivity were demonstrated with both statistical approaches, and most prominently in the grey matter (Fig. 3). Affected regions comprised the left and right caudates and thalami, hippocampi and parahippocampal gyri, the insular cortices, anterior and posterior cingulate gyri, the paracingulate gyri and sub-callosal cortices, as well as the frontal operculum and cerebellum. All diffusivity metrics were found to increase significantly bilaterally in the precentral gyri, in the right amygdala, left temporal fusiform gyrus, and the body of the corpus callosum line with time from baseline, though not significantly with ALSFRS-R decline. Axial diffusivity was shown to decrease in a small region within the left CST linearly over time. A small area of FA *increase* was noted for a region of the left posterior limb of the internal capsule in relation to ALSFRS-R decline.

### 3.4 Changes in resting state functional connectivity

**Fig. 4:**
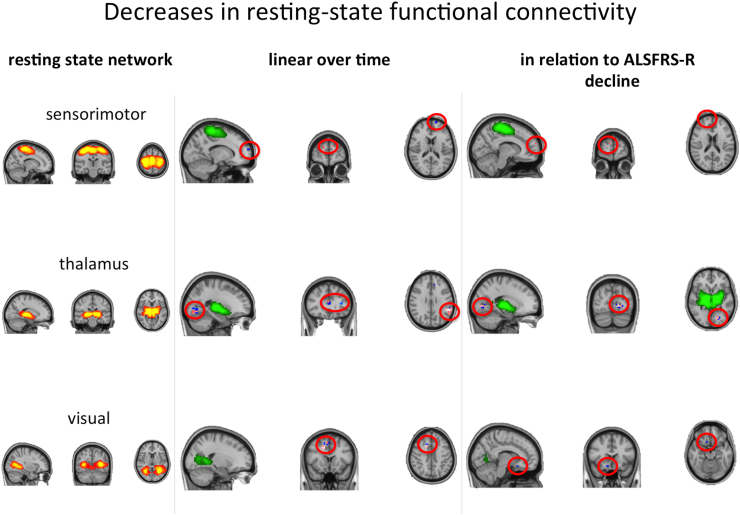
Progressive decreases in resting state functional connectivity with the sensorimotor, thalamic and visual resting state networks (RSN, shown thresholded at z > 3 in red-yellow in the left panel, shown in green otherwise) over time and in relation to ALSFRS-R decline. Clusters of significant change (encircled in red for improved visibility) are overlaid onto the respective RSN (green), as well as the mean filtered functional image in MNI space in the middle and right panel. Blue-lightblue – results without VBM regressor. Pink – results with VBM regressor.

**Fig. 5:**
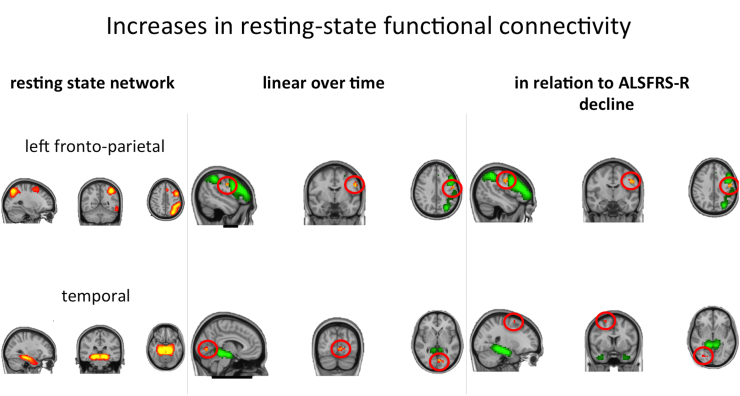
Progressive increases in resting state functional connectivity with the left fronto-parietal and the temporal resting state networks (RSN, shown thresholded at z > 3 in red-yellow in the left panel, shown in green otherwise) over time and in relation to ALSFRS-R decline. Clusters of significant change (encircled in red for better visibility) are overlaid onto the respective RSN (green), as well as the mean filtered functional image in MNI space in the middle and right panel. Significant results are shown in red-yellow for the analyses without VBM regressor (results for the analyses with VBM regressor are virtually the same and therefore not explicitly shown). (For interpretation of the references to color in this figure legend, the reader is referred to the web version of this article.)

Rs-fMRI analysis revealed FC decreases between the sensorimotor resting state network and the frontal pole, and between a network comprising both thalami and an area in the visual cortex in relation to both time and ALSFRS-R decline (Fig. 4). FC increases between the left fronto-parietal network and a region in the left primary motor cortex were seen for both statistical approaches (Fig. 5).

## 4 Discussion

Over a more extended observation period, involving serial MRI data over two years, the concept of ALS as a disorder involving progressive and widespread structural and functional brain changes was further reinforced. This cohort was modest in size and necessarily biased to slowly-progressive phenotypes physically more able to tolerate serial MRI scans. Widespread changes were nonetheless evident, including novel longitudinal functional connectivity insights.

### 4.1 Grey matter atrophy

The observed progressive grey matter atrophy common to both statistical approaches in the precentral gyri and posterior cingulate cortex, as well as the local atrophy demonstrated for basal ganglia structures, hippocampus and amygdala are in line with previous findings. Comparing data from two time points at least six months apart in a larger patient cohort using VBM, widespread grey matter volume decreases in motor and frontotemporal regions, the thalami and caudate heads bilaterally were observed (Menke et al., 2014). A prior study that investigated ALS-related grey matter atrophy over an average interval of nine months using tensor-based morphometry (TBM), reported a significant progression of atrophy in the left premotor cortex and in the right putamen and caudate nucleus (Agosta et al., 2009), while another demonstrated both cortical thinning and grey matter volume loss of the precentral gyri over an average interval of 1.26 years (Kwan et al., 2012).

A VBM study with six-month follow-up revealed widespread volume decreases in grey matter, particularly in the bilateral frontal and temporal lobes (Senda et al., 2011). In relation to involvement of the hippocampus (where the ALS molecular signature was long-since noted (Wightman et al., 1992)), a study which assessed ALS-related change in the volume of subcortical grey matter structures over an interval of on average 5.5 months revealed significant shrinkage of the right cornu ammonis 2/3 and cornu ammonis 4/dentate gyrus (Westeneng et al., 2015). However, another study that incorporated data from more than two time points (three over a mean total interval of six months), revealed no progressive grey matter atrophy based on VBM, cortical thickness analysis, and volume analysis of deep grey matter structures (Cardenas-Blanco et al., 2016).

### 4.2 Grey matter DTI changes

In addition to *atrophy* we observed widespread progressive *DTI changes* in the grey matter, particularly increases in mean, axial and radial diffusivity, in the thalami and caudate nuclei, the hippocampi and parahippocampal gyri, the insular cortices, anterior and posterior cingulate gyri, the paracingulate gyri and sub-callosal cortices, as well as the frontal operculum and cerebellar grey matter. This is consistent with two other longitudinal MRI studies that investigated DTI metrics in grey matter over time in MND, revealing extensive FA reductions in the thalamus, cingulum, hippocampal formation (van der Graaff et al., 2011), as well as frontal areas and the cerebellum (Keil et al., 2012) over time. While FA reductions and diffusivity increases of diffusivity in the grey matter are, and the potentially confounded by the effects of ageing (are inherently and particularly difficult to disentangle within (particularly in iron-rich deep grey matter structures), ageing has been explicitly been reported to have little effect on FA and diffusivity metrics in the thalamus (Pfefferbaum et al., 2010), so that alterations to this structure may therefore be particularly salient in appraisal of ALS pathology.

### 4.3 White matter DTI changes

We observed DTI changes for both statistical approaches in white matter tracts commonly reported to be affected in both cross-sectional and longitudinal studies in ALS, most prominently the superior longitudinal fasciculus, corticospinal tract and corpus callosum. While the pattern of white matter DTI changes over time is less consistently established, several studies have observed changes in similar regions between baseline and follow-up scans over intervals of 6 months, employing either region-of-interest approaches, TBSS analysis, or whole-brain analyses of DTI maps. One study combined tractography of the CST with whole-brain voxel-based analysis to investigate progressive white matter changes between baseline and six month follow up, revealing decreased FA, averaged across both CSTs, in limb-onset ALS (van der Graaff et al., 2011). More detailed FA profiles along the CST differed according to clinical phenotype. Between the two time points they observed that in bulbar-onset ALS, FA decreased significantly in the cerebral peduncle, caudal part of the posterior limb of the internal capsule and subcortical white matter, while in limb-onset ALS, FA decreased significantly in the medulla oblongata, cerebral peduncle, caudal part of the posterior limb of the internal capsule and subcortical white matter. Additionally, a whole brain voxel-wise comparison of baseline and follow-up FA maps, revealed extensive FA reductions in patients with bulbar-onset, comprising the white matter underneath the primary motor and sensory cortex, as well as the body and genu of the corpus callosum. In limb-onset patients, FA reduction over time was found throughout the corticospinal tract, in the body of the corpus callosum, in the white matter underneath primary the sensory cortex and anterior temporal pole, right thalamus and cingulum, and left optic radiations. Being one of few studies to include longitudinal follow up investigation in healthy controls, reassuringly none of the reported analysis approaches revealed any FA changes over time in the control sample.

A similar study investigating both FA and MD changes in ROIs of the CST showed a significant decrease of FA, but not MD, in the right superior CST (Zhang et al., 2011). This finding was further corroborated by whole brain voxel-wise analysis (Keil et al., 2012). We previously compared only two time points using TBSS, and demonstrated only a significant increase in axial diffusivity in the posterior limb of the left internal capsule (Menke et al., 2012). A subsequent enlarged cohort revealed similar results, demonstrating minor axial diffusivity increases in the left CST and increases for axial and mean diffusivity in a corpus callosum region of interest (Menke et al., 2014). Two additional studies have strengthened the evidence for progressive DTI changes in the CST and the corpus callosum, one demonstrating a significant linear decline of CST FA over three time points (Cardenas-Blanco et al., 2016), and a TBSS study revealing progressive increase of axial and mean diffusivity in the corpus callosum (de Albuquerque et al., 2017).

### 4.4 Progressive changes in resting state functional connectivity

This study demonstrated a mixed picture of FC decreases and increases between motor and non-motor cortical regions in relation to both time from baseline and ALSFRS-R decline. When interpreted in conjunction with the progressive loss of structural integrity, it is concluded broadly to reflect ALS as a progressive disintegration of motor and frontotemporal projection networks. A number of cross-sectional rs-fMRI studies investigating differences between ALS patients and controls have revealed functional connectivity abnormalities involving the sensorimotor network. Some reported predominantly reduced FC (Mohammadi et al., 2009, Jelsone-Swain et al., 2010, Tedeschi et al., 2012, Schmidt et al., 2014), others a mixed picture (Agosta et al., 2013, Chenji et al., 2016). Such changes may shed light on pathophysiological mechanisms and the balance of compensatory versus pathogenic connectivity changes as the ALS progresses. The symptomatic phase of ALS is characterised by cortical hyperexcitability (Vucic et al., 2011), which has obvious resonance with the long-standing concept of excessive glutamatergic stimulation in the pathogenesis of ALS (Rothstein et al., 1992), and by which the drug Riluzole is thought to exert its disease-modifying effect. However, some of the earliest functional neuroimaging studies hypothesized that the widened area of cortical activation seen in response to a motor task in ALS patients might specifically reflect loss of local inhibitory interneuronal circuits (Kew et al., 1993), for which there is a range of evidence (Turner and Kiernan, 2012). An acute recruitment of contralateral hemispheric brain regions is well-recognised after stroke (Calautti and Baron, 2003), therefore FC changes in ALS, whether as a result of local interneuronal circuit changes or not, might be similarly best considered as compensatory in relation to loss of motor cortical integrity. However, the one rs-fMRI study that considered FC specifically within the regions of structural disconnection in ALS, found *increases* in FC to correlate with faster rates of progression, raising the possibility of a potentially more directly pathogenic process underlying increases in FC (Douaud et al., 2011).

## 5 Conclusion

The observation of MRI changes over a more prolonged period demonstrates an ongoing brain-based pathological process in ALS, involving widespread structural and functional changes in the absence of major cognitive impairment. Grey matter MRI measures, including those from DTI, appear to have most promise as candidate surrogate markers of disease progression. Functional connectivity measures need further refinement and standardization, but have particular appeal as early proof-of-principle markers to assess therapeutic targeting.
